# Transactions of the American Medical Association

**Published:** 1860-08

**Authors:** 


### FROM TRANSACTIONS OF THE AMERICAN MEDICAL ASSOCIATION.

The Committee of Conference appointed to confer with the Committee of Medical Teachers offered the following resolutions for adoption by the Association:

*Resolved*, That it is the duty of medical colleges to require of every candidate for the degree of Doctor of Medicine, certificates of study during the full period of three years, under the direction of a regular practitioner of medicine, recognized by the American Medical Association, who shall certify, under his own hand, as to an attendance on two full courses of lectures, with an interval of at least three months between the termination of the first and the commencement of the second course.

*Resolved*, That every medical college shall keep a volume, in which every medical student presenting himself shall enter his name, his age, the period of his commencing the study of medicine, any diploma he may have received in evidence of previous education, with the name of the college or school from which he received such diploma, and the name of the preceptor with whom he has been studying.

*Reolved*, That hospital clinical instruction constitutes a necessary part of medical education, and every candidate should be required to have attended such instruction regularly for a period of not less than four months.

*Resolved*, That the professors of every medical college should recommend to their trustees, or board of managers, the adoption of a rule authorizing them to allow the attendance of two or three delegates from the State Medical Society, at all examinations of candidates for the degree of the doctorate, and accord to these delegates a vote on the question of recommending such candidates for a degree.

*Resolved*, That every State Society be recommended to choose proper delegates, at its annual meeting, to attend the examination of candidates for the degree of M. D., at all the medical colleges within their respective States.

*Resolved*, That this Association will not recognize as a regular organization any college which does not require evidence of suitable preliminary education from all applicants for collegiate medical instruction.

*Resolved*, That we commend the use of all proper efforts, by which the attention of persons of means and liberal disposition, is well as legislative bodies, shall be directed to the propriety of endowing such medical colleges, and professorships thereof, as shall be recognized by the Association.

*Resolved*, That this Association recognize as a regularly organized medical college, one which has been represented at any meeting of the Association, and which complies with the preceding rules and directions.

*Resolved*, That this Association recognize as regular practitioners of medicine, all who have been members of this Association, and have not forfeited their rights and privileges, and all members of State and County Societies in full standing:

The report was received and taken up by sections. When the first resolution came up, a motion was made to amend, by striking out that part requiring an interval of three months to elapse between the termination of the first course and the commencement of the second; the objection being that the resolution, if adopted as offered, would do an injustice to summer schools, whose sessions would have to begin three months after the closure of the winter sessions, in order to graduate students, thus throwing the session into July, August, and September, and crowding upon the next winter session; and that such a course would drive students altogether from the summer schools.

Dr. McDowell, of Missouri, spoke in strong terms against the amendment. He despised the plan of some professors, who teaching at a winter school in the South, immediately the winter session closes, bring their half-fledged brood to a Northern summer school, and there delivering a *second* course of lectures, foist their hastily hatched students upon the medical profession. He was entirely opposed to the practice of pushing and forcing which was becoming so rampant.

The discussion was further participated in by Drs. Shattuck, of Boston; Austin Flint, New York; Brodie, of Mich.; Palmer, of Mich.; Nourse, of Maine; Atlee, of Pa., and others.

Dr. John L. Atlee, of Philadelphia, would rather increase the interval to six months. Nor did he want, as others suggested, to leave the matter to the discretion of the professors in the different schools. His experience proved that a man never becomes a thorough and proper student of medicine until *after he has attended his first course of lectures;* for he then learns what is required, and how he should direct his studies so as to profit by them; and as he studies in the interval between the collegiate courses, the demonstrations of the previous winter reappear to him as he reads his text books, giving an interest to the study he could have sought for in vain before he had attended a course of lectures. He would rather have the course of instruction lengthened to eight or nine months, and have a less number of daily lectures. He wanted no student to have credit for attendance on more than one full course in one year, no matter how many regular courses he may have attended, and he moved to amend the amendment by striking out "an interval of three months," etc., and inserting, "and no student shall be credited for having attended more than one full course of lectures in any one year."

A motion of Dr. Bennett, of Danberry, Conn., to lay the whole matter on the table, was lost.

Dr. Johnson, of Mo., did not want the courses to follow too closely on each other. Apart from other considerations, such a course would crowd the student too much, and overtax his powers of physical and mental endurance. Students required time for relaxation, whether they wanted it or not; and therefore he was in favor of a considerable interval between the courses, and only one course in a year.

Dr. Worthington Hooker, of New Haven, Conn., thought there was not too much actual instruction being crowded together that was to be avoided, but rather too much lecturing, which brings different subjects in too close connection before the minds of the students, and taxes their energies too much, and therefore he did not want the courses crowded on each other; nor did he want the lectures to be as crowded as they usually are. He stated that in the medical department of Yale College, it was customary to make a distinction in the ability for receiving instruction, between medical students who had received the advantages of a previous classical education, and those who had not enjoyed this privilege; and that they considered classical students full one year in advance of the others, and that they made this distinction in their favor regarding the length of time required to be devoted to the special study of medicine. They only required two years' application from classical students, while they exacted three years' study from all others. He firmly believed that a difference of one year should be made, but he would prefer the course of application to be extended one year longer in each case, thus making it three and four years' study, instead of two and three, as it now is.

Dr. Atlee was here allowed to alter his amendment so it should read, "and no student shall attend a second course of lectures until a year shall have elapsed since the commencement of the first course."

Dr. Reese, of N. Y., remarked, that as the winter schools closed in March, and the summer schools could not, by the resolution, begin in June, this would force students to employ in study the months of July and August, which is the general period of relaxation from labor; and thus, virtually, in a great measure, prevent graduation at the summer schools.

After some more discussion, the amendment of Dr. Atlee was adopted, when a motion was made by the opponents of the amendment, to lay the resolution on the table. This motion was lost, and finally, the resolution as amended, was adopted.

Second resolution, adopted.

Third resolution, adopted.

The fourth resolution was amended by Dr. McCaw, requiring "that in those States where there are regular State Medical Societies, the delegates elected to be present at the examinations of candidates for the degree of M. D., in all the medical schools of the State, should be selected from the members of the State Society; in those States where there are no societies, the selection is to be made from members of the profession in good standing."

Dr. John L. Atlee wanted no representation of examiners from any except State Medical Societies, which would force those States where it does not now exist to organize State Medical Societies; and, therefore, he opposed the amendment, though he favored the original resolution.

Dr. Storer, of Mass., wished no restrictions to be placed on medical schools.

Dr. Mussey, of La., wanted to have a resolution passed, providing for the examination of teachers before they were elected to professorships, and to pass no men but those who were known to be favorable to the ideas of the Association regarding medical instruction.

Here a tumultuous clamor arose, during which the amendment was withdrawn, and the previous question called for and sustained; when the resolution was, upon call, again read, and finally adopted as originally reported.

The fifth resolution gave rise to a great deal of discussion as to the propriety and the right of placing medical schools under the censorship of State Medical Societies.

Dr. Timothy Childs, of Berkshire, Mass., stated, that forty years ago he called for a board of examiners to be present at all examinations for a degree, and that he had never ceased to urge the propriety of so doing. He had never passed a student without such a supervision.

He stated that he was the first man to introduce into medical colleges a professorship on pathology, and he was always in favor of enhancing the dignity and worth of his profession; and as long as he was able to raise his voice, he would oppose to the utmost, all those who attempt to lower the standard of medical excellence, regardless of the motives that prompt them to do so.

Dr. Worthington Hooker, of New Haven, Conn., explained that Yale College, further back than forty years ago, had, of its own accord, adopted the plan contained in the resolution under consideration; and, during his connection with the college, there had not been one whisper of disapprobation regarding it. There was harmony between the State Medical Society and the institution, which feels the genial effects of that harmony, which gives it its strength and position.

He thought that all medical colleges should be closely watched by the State Medical Societies of their respective States.

The resolution was adopted.

The sixth, seventh, and eighth resolutions were adopted.

The ninth resolution was, after some little discussion, on motion, laid upon the table.

The whole report was then adopted and referred to the Committee on Publication, for publication in the forthcoming volume of Transactions.

